# Germination and the Early Stages of Seedling Development in *Brachypodium distachyon*

**DOI:** 10.3390/ijms19102916

**Published:** 2018-09-25

**Authors:** Elzbieta Wolny, Alexander Betekhtin, Magdalena Rojek, Agnieszka Braszewska-Zalewska, Joanna Lusinska, Robert Hasterok

**Affiliations:** Department of Plant Anatomy and Cytology, Faculty of Biology and Environmental Protection, University of Silesia in Katowice, 28 Jagiellonska Street, 40-032 Katowice, Poland; alexander.betekhtin@us.edu.pl (A.B.); magdalena.rojek@us.edu.pl (M.R.); agnieszka.braszewska-zalewska@us.edu.pl (A.B.-Z.); jlusinska@us.edu.pl (J.L.); robert.hasterok@us.edu.pl (R.H.)

**Keywords:** *Brachypodium distachyon* embryo, cell cycle, EdU, germination, replication

## Abstract

Successful germination and seedling development are crucial steps in the growth of a new plant. In this study, we investigated the course of the cell cycle during germination in relation to grain hydration in the model grass *Brachypodium distachyon* (Brachypodium) for the first time. Flow cytometry was performed to monitor the cell cycle progression during germination and to estimate DNA content in embryo tissues. The analyses of whole zygotic embryos revealed that the relative DNA content was 2C, 4C, 8C, and 16C. Endoreplicated nuclei were detected in the scutellum and coleorhiza cells, whereas the rest of the embryo tissues only had nuclei with a 2C and 4C DNA content. This study was accompanied by a spatiotemporal profile analysis of the DNA synthetic activity in the organs of Brachypodium embryos during germination using EdU labelling. Upon imbibition, nuclear DNA replication was initiated in the radicle within 11 h and subsequently spread towards the plumule. The first EdU-labelled prophases were observed after 14 h of imbibition. Analysis of selected genes that are involved in the regulation of the cell cycle, such as those encoding cyclin-dependent kinases and cyclins, demonstrated an increase in their expression profiles.

## 1. Introduction

Seed germination is considered to be the initiation of the first developmental phase in the lifecycle of higher plants and is followed by the postgerminative growth of the seedling [1]. A seed starts to germinate in favourable conditions in response to environmental stimuli such as light, temperature, soil components (especially nitrate), and the molecular mechanisms of a response that have been well characterised [2]. Germination is a complex process during which the mature seed resumes growth and shifts from a maturation- to germination-driven programme of development and subsequent seedling growth [1,3]. By definition, the germination of a seed starts with the uptake of water and is completed when the radicle protrudes from the covering structures [4]. In the case of the germination of a monocotyledonous plant seed, the coleorhiza is the first part to grow out of the seed coat, whereas during the germination of a dicotyledonous plant seed, the radicle grows out of the seed coat first [5]. In both groups, the progress of germination is strictly related with the water uptake rate. Initially, there is a rapid imbibition of water by a dry seed (phase I) until the seed tissues are fully hydrated. This is followed by a limited water uptake during phase II, whereas in phase III, there is an increase of water uptake that is related to the completion of germination. The most important is phase II, which is associated with various cellular and biochemical events such as DNA repair and the translation of stored as well as newly synthesised mRNAs. Phase II is characterised by both increased metabolic and cellular activity. At the germination stage, the decision of embryo cells to re-enter the cell cycle or to remain arrested is crucial in determining seedling formation [6,7]. The cell cycle, which is arrested in a quiescent seed, is reversed during germination. The replication of DNA is a relatively late event in seedling formation, and it occurs after a seed imbibes water at the end of phase II. In most species, the protrusion of the radicle does not require mitotic activity [8], although in tomato, the activation of cell division has been found to occur before the protrusion of the radicle [9].

At the germination stage, activation of the cell cycle is crucial to seedling formation [7]. The cell cycle is a coordinated recurrent series of events that occur between the ends of subsequent cell divisions, by which the cellular material is duplicated and divided into daughter cells. The progression of the cell cycle is controlled via reversible phosphorylation by protein kinases and phosphatases and by the activity of cyclin-dependent kinases (CDK) and their activating subunits, cyclins (CYC). In plants, there are five groups of CDK (CDKA to CDKE). Kinases from the CDKA group exhibit a PSTAIRE motif in their amino acid sequence that is involved in their interaction with cyclins, whereas CDKBs contain another specific protein motif, PPTA/TLRE. CDKAs play a role in both the G1-to-S and G2-to-M transition, whereas the proteins from the CDKB group are required to progress through mitosis [8,10]. Plant cyclins have been classified into five groups: A, B, C, D, and H. The A-type cyclins are expressed during the S, G2, and M phases, while the B-type cyclins accumulate during the G2-M phases and regulate the entry into the M phase. The D-type cyclins control the progression from the G1-to-S phase in response to external growth signals, but some of them are also involved in the G2-to-M transition [11].

Seedling growth from the embryo is primarily driven by cell expansion in the embryonic axis. Following germination, most of the future growth of a plant is dependent on the cell divisions that occur in both the root and the shoot meristems within the mature plant embryo. Activation of the embryo root meristem is necessary for the initiation of root growth and is a key component of the establishment of a seedling [12]. The activation of the mitotic cell cycle has previously been demonstrated to occur in the shoot and root meristems during the final stages of seed germination and to be dependent on the hormone gibberellic acid (GA). Many studies have identified crucial roles for GA and abscisic acid (ABA) during seed germination. The application of exogenous ABA inhibits germination and *Arabidopsis thaliana* (Arabidopsis) mutants that are deficient in ABA biosynthesis or signalling have an enhanced germination efficiency. Conversely, GA also promotes seed germination. GA-deficient mutants show a delay or absence of seed germination [13]. Moreover, other hormones, i.e. ethylene, brassinosteroids, cytokinins and auxins, also influence germination [1]. Apart from phytohormones, other germination stimulants are known, some of which are important environmental sensors. Among them, the nitrogen-containing compounds (NO; NO_2_^−^; NO_3_^−^), which stimulate germination, as well as reactive oxygen species (ROS) exert control over germination, most likely together with NO, in order to regulate the catabolism of ABA and the biosynthesis of GA during seed imbibition. ROS and phytohormones have also been suggested to be involved in modulating radicle growth during Arabidopsis germination [1,14].

*Brachypodium distachyon* (Brachypodium) has been proposed by Draper et al. [15] as a model organism due to its close relationship with economically important temperate cereals and forage grasses combined with many other favourable attributes, such as its very small nuclear genome, simple growth requirements, small stature, and rapid annual cycling. A Brachypodium genome sequence is available and diverse molecular and genetic tools have been developed for its transformation, mutagenesis, and gene mapping. Accessions that have been collected from its ancestral range show a surprising degree of phenotypic variation in many traits, including those that are implicated in the domestication of cereals. Thus, Brachypodium can be a useful model for investigating various problems of grass biology [16,17], not only in research on the cell wall composition [18,19,20] or Brachypodium–pathogen interactions [21,22], but also to study the life stage of the seed-to-seedling transition. A detailed characterisation of the germination process and seedling growth has been carried out in Arabidopsis, which is a model species for dicotyledonous plants [7] as well as in domesticated grass species such as barley [8,23] and maize [24,25]. In this study, the events that occur during seed germination and early seedling development in Brachypodium, which is a wild grass, were analysed at the cellular and molecular levels.

## 2. Results

### 2.1. Germination Characteristics

**Figure 1:**
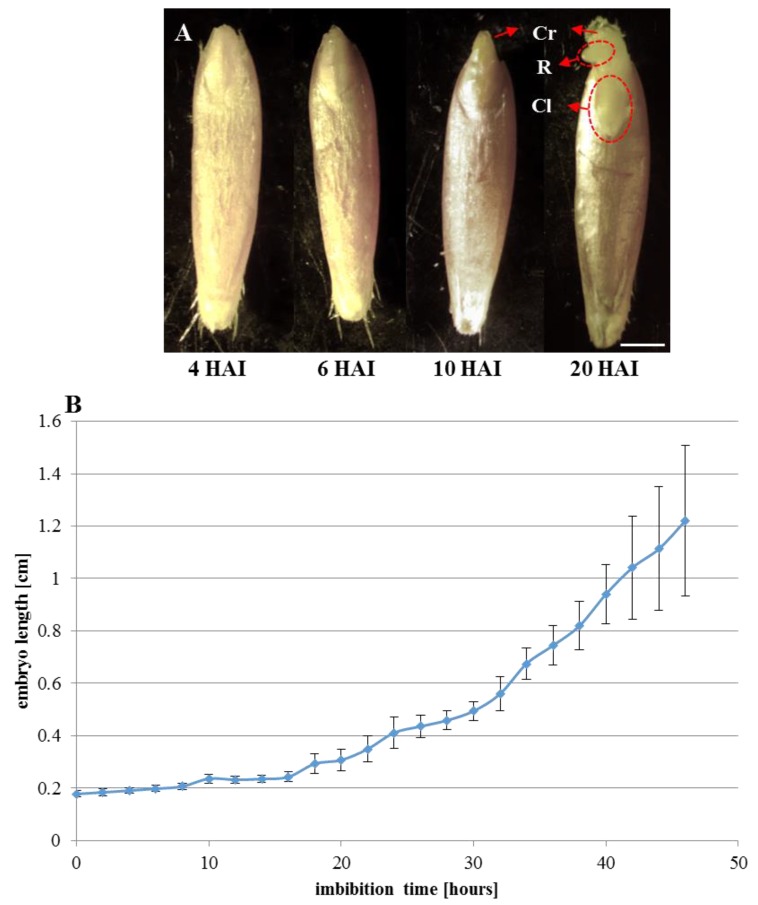
*Brachypodium distachyon* (Brachypodium) grains at key time points of imbibition (**A**) and the progress of Brachypodium embryo size growth during 46 h of imbibition (**B**), data are means ± SE. Abbreviations: Cr—coleorhiza, R—radicle, Cl—coleoptile. Scale bar represents 1 mm.

Using the appearance of the coleorhiza and root emergence as indicators, germination was monitored over a 46-h period of the seedling development (Figure 1). Caryopsis covering structures, i.e., seed coat and fruit coat, were disrupted at 6 h after imbibition (HAI) in most of the seeds (Figure 1A). In the majority of the seeds, the coleorhiza appeared at 8 HAI, while at 10 HAI, almost all of the seeds had a coleorhiza that was already visible. The primary root emergence and coleoptile growth were the next steps in the Brachypodium seedling development. Primary roots appeared at 20 HAI (Figure 1A) in some seedlings, while at 24 HAI, almost all of the seedlings had visible roots. The measurements of the embryo length during germination and growth at various time points are presented in Figure 1B. The embryo size in dry seeds was estimated to be approx. 0.2 cm in length. During the first 16 h of imbibition, the size of the Brachypodium embryo increased only slightly. Intense embryo growth began at 18 HAI, and at 24 HAI, the Brachypodium embryos had doubled their length.

### 2.2. Nuclear DNA Content and Cell Cycle Analysis in Brachypodium Embryos

**Figure 2:**
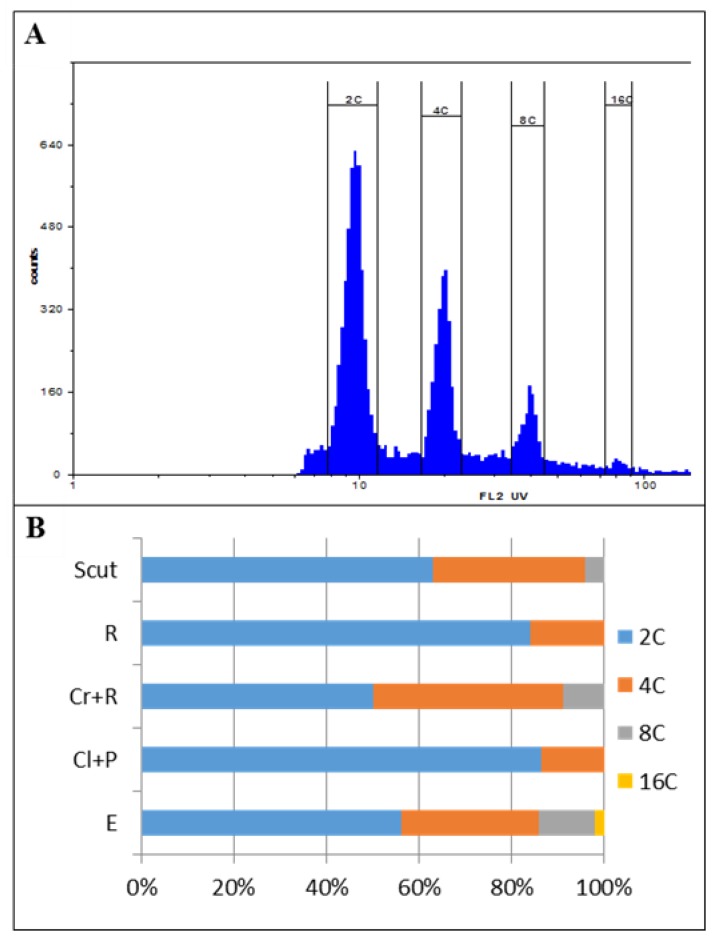
Flow cytometry analysis of DNA content pattern in the Brachypodium embryos that were isolated at 4 h after imbibition (HAI) (**A**). Histogram showing the ploidy distribution in whole embryos. (**B**) Percentage of cells with a 2C, 4C, 8C, and 16C DNA content in a whole embryo (E) and in specific embryo organs, (Scut) scutellum, (R) radicle, (Cr + R) coleorhiza and radicle, (Cl + P) coleoptile with a shoot apex and primary leaves.

Flow cytometry is a fast and suitable method for analysing DNA content in different plant organs. Nuclei stained with DAPI can be grouped based on their fluorescence intensity. To determine the DNA content pattern in Brachypodium embryos, whole embryos were selected at 4 HAI. Flow cytometry analysis revealed the presence of four peaks that corresponded to the 2C, 4C, 8C, and 16C DNA content (Figure 2A). The dominant fraction comprised nuclei with a 2C and 4C DNA content (44.8% and 23.62%, respectively). The percentage of nuclei with an 8C and 16C DNA content was lower and was observed at a frequency of 9.6% and 1.64%, respectively (Figure 2B). The presence of nuclei with an 8C and 16C DNA content indicates the occurrence of endoreduplication in Brachypodium embryo tissues. Flow cytometry analysis for the isolated embryo parts (Figure 2B) showed that the process of endoreduplication was primarily restricted to the scutellum and coleorhiza and the percentage of this type of nuclei was 2.96 and 6.29, respectively (Figure 2B). On the other hand, the primary root and the sample that had been prepared from the plumule and coleoptile tissues had more than 80% of 2C DNA nuclei, thus indicating that the majority of the cells were arrested at the G1 phase of the cell cycle (Figure 2B). Nuclei with 16C DNA were not detected in the analyses of specific embryo organs due to their very low number and the lack of visible peaks. 

Flow cytometry was also performed to monitor the progression of the cell cycle during seed germination. Embryos were analysed from 4 to 24 HAI. Based on the histograms, the percentage of cell nuclei in specific phases (G1, S, G2) was determined using the Cell Cycle Analysis function in FlowMax software, which is summarised in Table 1. 

**Figure 3:**
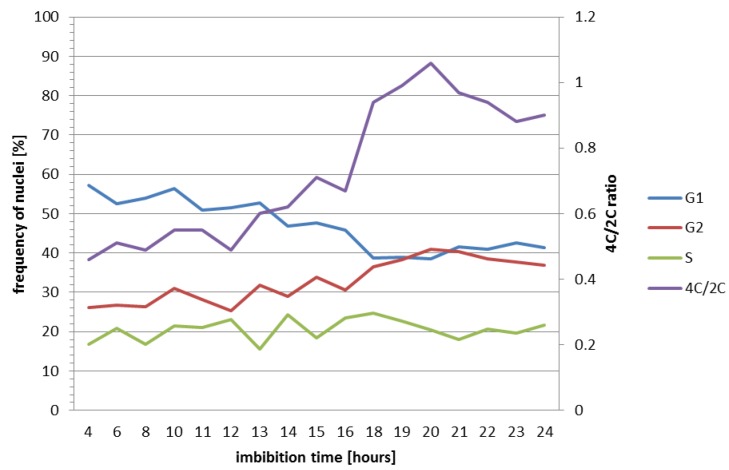
Percentage of nuclei in the G1, S, and G2 phases and the 4C/2C ratio in the cells from Brachypodium embryos during the first 24 h of growth.

For better clarity, the correlation data are also presented in a diagram (Figure 3). We observed that during the process of seed germination, the number of cell nuclei with 4C DNA content increased to 41% at 20 HAI. At the same time, a decrease in the number of cell nuclei with 2C DNA content from 57% at 4 HAI to 38% at 18–20 HAI was observed. The percentage of nuclei in the S phase was not constant and fluctuated during seed germination. The lowest number of cell nuclei in the S phase was observed at 4 and 13 HAI (16.7% and 15.54%, respectively), and the highest was at 14 and 18 HAI (24.24% and 24.77%, respectively) (Table 1). The ratio that was calculated between the nuclei with 4C and 2C DNA content showed an increase during the embryo growth, which was particularly high between 16 and 20 HAI (Table 1; Figure 3). The whole mature Brachypodium embryo displays a large proportion of cells with nuclei having 4C DNA content (Figure 2). These cells can be arrested in the G2 phase of the cell cycle before cell division but can also represent endoreplicating cells.

### 2.3. Distribution of DNA Synthesis

**Figure 4:**
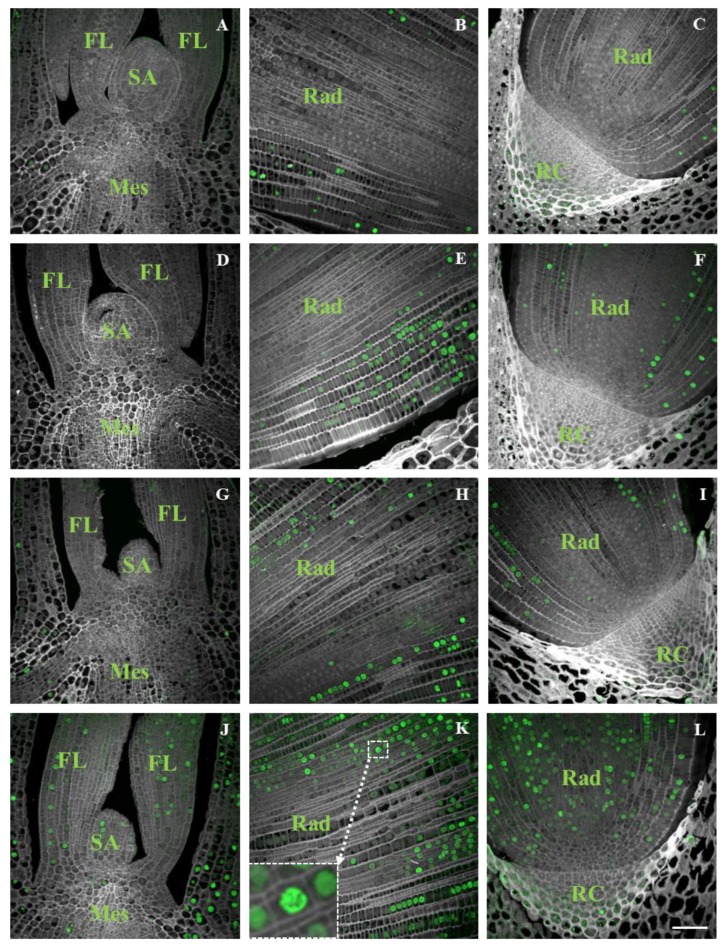
Detection of EdU (green fluorescence) incorporated into actively replicating nuclei in Brachypodium embryos during imbibition. Fragments of cross sections through embryos 11 HAI (**A**–**C**), 12 HAI (**D**–**F**), 13 HAI (**G**–**I**), and 14 HAI (**J**–**L**). Cross sections through the shoot apex (**A**,**D**,**G**,**J**), radicle (**B**,**E**,**H**,**K**), and radicle with a visible root cap (**C**,**F**,**I**,**L**). Inset in (**K**) presents an enlargement with the nucleus at prophase. Abbreviations: SA—shoot apex, FL—first leaf, Mes—mesocotyl, Rad—radicle, RC—root cap. The slides were counterstained with DAPI (grey fluorescence). The grey colour of cell walls was caused by their autofluorescence. Scale bar represents 20 µm; all photomicrographs were taken at the same magnification.

DNA replication, as detected by flow cytometry, was compared with the analysis of DNA synthesis that was visualised by the incorporation of EdU into the actively replicating nuclei. The presence of EdU was not found in the nuclei of embryos at 4–10 HAI but was observed in increasing levels in the nuclei of embryos at 11–14 HAI (Figure 4).

Based on these results, we assume that the replication in the Brachypodium embryo tissues started at approx. 12 HAI. Initially, most of the EdU-labelled nuclei occurred only in the radicle tip (Figure 4E,F). In the embryos at 13 HAI, labelled nuclei were also visible in the coleoptile (Figure 4G), and at 14 HAI, EdU signals were visible in the SAM and primary leaves (Figure 4J). Between 11 and 14 HAI, the number of labelled nuclei increased significantly, especially in the radicle. The first labelled nuclei were observed in the cortex cells and epidermis layers of the radicle tip and subsequently in the central cylinder and root cap cells (Figure 4C,F,I,L). We did not observe EdU signals in the cell nuclei of the scutellum in any of tissue sections regardless of developmental stage being analysed. Nuclei at the prophase of mitosis were observed in a few cells of the radicle at 14 HAI (Figure 4K, inset).

### 2.4. Analysis of Cell Cycle Gene Transcript Profiles Using Reverse-Transcription PCR

**Figure 5:**
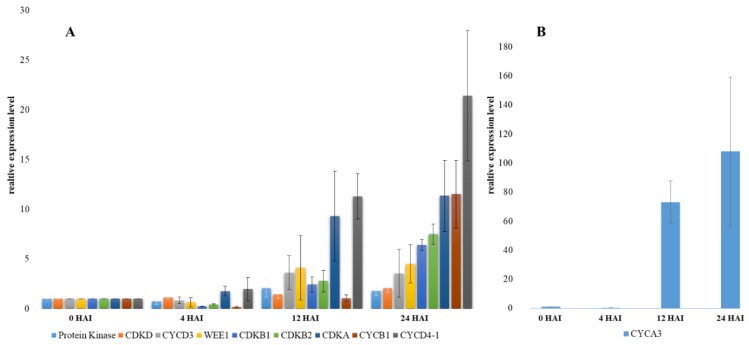
Expression of the *CDKA*, *CDKB1*, *CDKB2*, *CDKD*, *CYCB1*, *CYCD3*, *CYCD4-1*, *WEE1*, and *protein kinase* genes (**A**) and the *CYCA3* gene (**B**) that were involved in the cell cycle activity in Brachypodium embryos at 0, 4, 12, and 24 HAI. The relative expression levels were normalised to an internal control (AK437296, gene encoding for ubiquitin) and calibrated to the control (the embryos from dried seeds). The data from RT-PCR (mean ± SD of three replicates) are given in arbitrary units.

To evaluate any changes at the molecular level, the transcript profiles of the key cell cycle genes were analysed using RT-PCR. Four time points (0, 4, 12, and 24 HAI) during seed imbibition were selected, which covered the complete seed germination process. Complementary DNAs (cDNAs) were used as the templates for the PCR amplification with the appropriate primers (Table 2). Specific transcript accumulation patterns were identified for CDK (*CDKA*, *CDKB1*, *CDKB2*, *CDKD*) and cyclins (*CYCA3*, *CYCB1*, *CYCD3*, *CYCD4*) as well as for the regulatory gene *WEE1* (Figure 5). The gene-expression changes were significant in the case of most of CDK except for *CDKD*, the transcripts of which did not progressively accumulate during seed imbibition. The expression of *CDKA* increased abruptly between 4 and 12 HAI, whereas during the next 12 h, the transcript level did not increase considerably (Figure 5). Moreover, the expression of cyclins also increased. The expression levels of *CYCA3* and *CYCD4* increased rapidly during the first 12 HAI in contrast to *CYCB1*, the expression of which increased abruptly between 12 and 24 HAI. The expression of *protein kinase*, *CYCD3*, and the regulatory gene *WEE1* increased during the first 4 h of imbibition and did not change significantly during the subsequent hours of growth (Figure 5).

## 3. Discussion

Germination is a sequence of molecular events that transforms a heterotrophic embryo into a complex autotrophic organism and is a major developmental transition in the lifecycle of a plant that involves the concerted action of numerous genetic and physiological pathways and primarily consists of the resumption of embryo growth after the dormancy that is imposed during seed maturation. During the switch from dormancy to a growth mode, upon the imbibition of water, the cells of an embryo are sequentially activated [26]. In the present study, the successive development of the DNA synthetic activity was demonstrated in the embryonic cells of the germinating seeds of Brachypodium using flow cytometry and a Click-iT EdU assay. RT-PCR was used to characterise the expression of the main genes that are involved in the regulation of the cell cycle.

Studies of the cellular and molecular events that occur during plant embryo germination require a detailed analysis of the nuclear processes and the cell cycle parameters. There is no data on these processes, which occur during germination in Brachypodium. Although Brachypodium has been accepted as a model plant for molecular biological research in cereals and temperate grasses, its use in studies of the early stages of plant growth is limited. However, a comprehensive overview of the Brachypodium embryo and grain development was provided by Guillon et al. [27] and the research that was conducted by Opanowicz et al. [28] shed light on the development of the endosperm. Barrero et al. [29] provided an anatomical description of Brachypodium caryopsis and also studied grain dormancy. Later, studies on the global histone modifications and DNA methylation changes during embryonic and postembryonic growth in Brachypodium [30,31] and on the breeding system, caryopsis structure, and germination [32] in annual and perennial Brachypodium species were performed.

During germination, Brachypodium seeds showed distinctive characteristics of physiology and morphology. During imbibition, the dry seeds absorbed water, which resulted in an increase in seed volume that caused the seed coat tissues to be disrupted at 6 HAI. The coleorhiza generally emerged from the seeds after 10 h of water absorption and the primary root appeared within 24 h. In Brachypodium embryos at 4 HAI, flow cytometric analysis revealed that only 2C and 4C nuclei were present in the primary root and plumule, whereas cells with 8C DNA were detected in the coleorhiza and scutellum. The proportion of nuclei with a 2C and 4C DNA content was approx. 4:1 in the primary root and shoot, thus suggesting that the majority of the cells of both organs were at the G1 stage, while, for example, in the embryo cells of barley, 92% of the nuclei in the radicle tips were arrested in the G1 phase of the cell cycle. The same high percentage of nuclei with a 2C DNA content was also observed in tomato and pepper [33]. In other species, such as *Lactuca sativa* and *Pinus nigra*, only nuclei with a 2C DNA content were found in the radicle cells, while 4C and 8C DNA nuclei were observed in *Phaseolus vulgaris* and *Spinacia oleracea* [23,33]. In Brachypodium, endoreplicated nuclei with an 8C DNA content were only detected in the scutellum and coleorhiza. Due to the low percentage of nuclei with a 16C DNA content, we were unable to separate this class of nuclei during the flow cytometric analysis of the isolated organs of Brachypodium embryos. Endoreplicated nuclei with an 8C and 16C DNA content were identified in the parenchyma cells of the scutellum of *Triticum durum* [34]. The scutellum and aleurone layers play an essential role in the germination process by producing hydrolytic enzymes in order to mobilise the storage compounds of the starchy endosperm, which support early seedling development [35,36]. The scutellum also acts as a reserve that secretes, absorbs, and transfers nutrients, and the high level of endoreplication in this organ is probably due to its specific function. The coleorhiza covers the radicle and plays a major role in initiating dormancy by acting as a barrier to the emergence of the root. The catabolism of ABA occurs in the tissues that surround the root in the seed and the amount of ABA in the coleorhiza is a key factor in controlling dormancy and germination [37]. The flow cytometric analysis of Brachypodium embryos showed an increase in the 4C/2C ratio during imbibition. After 10 h, when coleorhiza had emerged, the ratio was 0.59. The highest (1.06) ratio was observed in the embryos at 20 HAI, when the primary root broke through the coleorhiza and the intensive growth of the root started, which was related to a high frequency of cell divisions. Similar results were obtained for barley, in which the ratio increased to 1.2–1.5 [23] during radicle growth. Moreover, a clear increase in the 4C DNA content along with a decrease in the population of cell nuclei with 2C DNA was observed at the moment the radicle emerged in Arabidopsis [7].

In our study, replicative DNA synthesis, as assessed by flow cytometry, was compared with the analyses of DNA synthetic activity using labelling with the thymidine analogue EdU. The nonexistence of DNA replication during the first 10 h of Brachypodium imbibition was demonstrated by the absence of the incorporation of EdU into the DNA. The replicating nuclei were primarily present in the cells of the cortex and in the epidermis layer of the root meristem of the embryos at 11 HAI. During the next few hours of imbibition, the number of replicating nuclei successively increased and they were visible in almost all of the organs of the embryos except the scutellum. Moreover, no DNA synthesis occurred in the nuclei of the quiescent centre. The timing of the replication activity in the Brachypodium embryos using EdU is consistent with the results that have been obtained from the flow cytometric analyses as well as with similar research that has been performed on cucumber [38] and tomato [9], whereas in root tips of white cabbage, the onset of DNA replication precedes root protrusion [7,39]. However, a large portion of cells in mature Brachypodium embryo has nuclei with 4C DNA content. During the first 24 HAI, there was little alternation in the percentage of endoreplicated nuclei. This might indicate that there are some cells with 4C DNA nuclei that represent the G1 phase of endoreplicated cells, while others are arrested in the G2 phase, which may suggest that cell divisions can occur in the absence of DNA replication and contribute to germination. Cell divisions have been found prior to the protrusion of the radicle in tobacco and tomato [9]. In barley, the application of hydroxyurea, which resulted in a blocking of the cell cycle, did not prevent germination but inhibited radicle growth [8]. This suggests that the initiation of the cell cycle may not be totally required for the early phases of germination. Cell divisions did not take place in coleorhiza and this tissue grows without cell division during germination, as was shown for barley [37], but we cannot rule out the possibility that radicle cells with 4C DNA may undergo mitotic divisions. The obtained results suggest that the activation of cell division is not involved in the emergence of the coleorhiza in Brachypodium, and the same observation was reported during the emergence of the radicle in Arabidopsis by Barroco et al. [7]. The first prophases were observed in a few of the meristematic root cells of the Brachypodium embryos at 14 HAI and had EdU signals. However, our results of RT-PCR analyses indicate an increase in *CDKA* and *CYCD4-1* expression levels at 4 HAI and the products of these genes are known to be involved in the control of G2-M transition [40].

The presented results demonstrate that major changes in the transcript levels of the CDK and CYC genes occurred in the embryo during germination. The embryos from three key stages (4, 12, and 24 HAI) as well as dry seeds (0 HAI) were selected for the RT-PCR analyses. All of the embryos at 12 HAI had a visible coleorhiza and the replication started at this time point, whereas after 24 h of growth, the seedlings had visible radicle. As was revealed by RT-PCR, the onset of the emergence of the coleorhiza and radicle was marked by the transcript accumulation profiles for most of the genes that were analysed except *CDKD*. This type of cyclin-dependent kinase as well as *CDKF* belong to the group of CDK-activating kinases and phosphorylates and activate all of the core CDK, i.e., *CDKA*, *CDKB1*, and *CDKB2*, thereby governing the progression of the cell cycle throughout the development of a plant [41,42]. The RT-PCR results showed an expression outbreak of *CYCB1* that coincided with the timing of the emergence of the radicle. In plants, the *CDKB* class shows the cell cycle regulated expression in the G2-to-M phase [43]. An analysis of gene expression using the *CYCB1;1-GUS* reporter construct revealed a patchy expression pattern in the dividing cells of Arabidopsis [44]. We observed a significant increase in the transcription levels of *CYCA3* and *CYCD4;1*. The level of *CYCD4;1* was higher in the embryos at 12 HAI than at 4 HAI, which is consistent with the increasing proportion of 4C nuclei. The expression of *CYCA3* was also found to increase abruptly at 12 HAI. Both cyclins are probably involved in the re-establishment of the cell cycle activity and preparation for the G1-to-S transition in Brachypodium. This observation is in agreement with the results of the EdU detection analyses, which indicated the induction of replication at this time point of Brachypodium germination. Similar findings were also observed for Arabidopsis [7] and barley [23]. The results presented here indicate that the cell cycle was initiated before the emergence of the coleorhiza, but it should be noted that transcription may not correlate with the onset of translation. However, the detection of EdU in the tissues of Brachypodium embryos clearly demonstrated the entrance into the S phase at approximately 12 HAI and the emergence of the radicle thereafter. 

## 4. Materials and Methods

### 4.1. Plant Material and Germination Assay

A community standard inbred line of Brachypodium Bd21 was used in this study. To exclude the impact of lemma on the germination rate, this structure was removed from the seeds prior to germination. Then, the seeds were placed on three layers of filter paper that had been soaked with distilled water in Petri dishes and germinated at 22–24 °C in the dark. For the germination assay, approximately 30 Brachypodium seeds were analysed. To calculate the average length of the embryonic axis, images of germinating seeds were recorded every 2 h starting at the 4th hour after the beginning of imbibition until hour 48 of seedling growth. The lengths of the embryo axis were measured using Image J (Wayne Rasband, National Institutes of Health, Bethesda, MD, USA).

### 4.2. Flow Cytometry

Flow cytometry was used to analyse ploidy and the cell cycle progression in Brachypodium embryos. To determine the general DNA content, whole embryos were selected at 4 HAI. For the analysis of the organ-specific DNA content, specific parts of the embryos were dissected using binoculars and needles, after which the samples that contained the individual scutellum, coleoptile with a shoot, and a primary root with coleorhiza were prepared. To determine the cell cycle progression, whole embryos from the germinating seeds at various time points starting at 4 HAI and ending at 24 HAI were used. Approximately 30–50 embryos were used for each analysis. After mechanical tissue fragmentation in a Nuclei Extraction Buffer (CyStain^®^ UV Precise P, 05-5002, Sysmex), the suspension of nuclei was filtered through a 30-µm nylon mesh to remove any large debris and then stained with a staining buffer containing DAPI and 1% betamercaptoethanol (CyStain^®^ UV Precise P, 05-5002, Sysmex). For all of the analyses, the samples were incubated for 1–2 min and analysed using a CyFlow Space flow cytometer (Sysmex, Kobe, Japan). with a 365-nm UV LED diode as the light source. The flow rate was adjusted to 20–40 nuclei per second. The results are presented on histograms using a logarithmic scale for the ploidy and a linear scale for the cell cycle analysis. To determine the cell cycle phases, software FloMax was used with the application of Cell Cycle Analysis.

### 4.3. EdU Detection on Embryo Cross Sections

The experiments with EdU, which is an indicator of DNA replication, were performed by incubating the seeds with this compound. The seeds were placed on filter paper that had been soaked with a 10-mM water solution of EdU to initiate their germination. The germinated seeds were fixed in a 4% paraformaldehyde solution in PBS at 4, 6, 8, 10, 11, 12, 13, and 14 HAI. The procedures of embedding the embryos in Steedman’s wax [45,46] and preparing the slides were done according to Wolny et al [30]. Prior to the EdU detection, the de-embedded slides were permeabilised with 0.5% Triton X-100 for 15 min and then washed in PBS at RT. The slides were incubated for 30 min at RT in an EdU reaction cocktail (Click-iT EdU Imaging Kits Alexa Fluor 488, Invitrogen, Carlsbad, CA, USA), which was prepared according to the manufacturer’s instructions. The slides were mounted and counterstained in Vectashield (Vector, Burlingame, CA, USA) containing 2 µg/mL of DAPI (Sigma-Aldrich, St. Louis, MO, USA). The fluorescence of the DAPI (excitation 405 nm, emission 425–475 nm) and Alexa 488 (excitation 488 nm, emission 500–600 nm) was registered using an Olympus FV1000 confocal system (Olympus, Tokyo, Japan) equipped with an Olympus IX81 inverted microscope (Olympus, Tokyo, Japan). Image processing operations were applied uniformly using an ImageJ package. For each of the stages that were studied, at least three embryos were randomly selected. Approximately 10 sections on the same slide were observed for each embryo. Photomicrographs were taken from sections from the middle part of an embryo at specific stages.

### 4.4. Gene Expression Analysis

RNA was isolated from dry seeds (0 HAI) as well as from the developing embryos/seedlings at 4, 12, and 24 HAI. The embryos and seedlings were isolated from the endosperm using tweezers, then frozen in liquid nitrogen and stored at −80 °C. For each extraction, approx. 100 embryos and seedlings that were ground in liquid nitrogen and a NucleoSpin RNA Plant and Fungi Kit (Macherey-Nagel, Duren, Germany) were used to isolate the total RNA. The RNA was purified using Qiagen RNase-Free DNase set (Qiagen, Venlo, Netherlands) and an RNeasy MinElute Cleanup Kit (Qiagen, Venlo, Netherlands). The concentration and quality of the isolated RNA was evaluated using an ND-1000 NanoDrop spectrophotometer (Thermo Scientific, Waltham, MA, USA). First-strand cDNA was produced using a Maxima H Minus First Strand cDNA Synthesis Kit (Thermo Scientific, Waltham, MA, USA). The primers that were relevant to the genes that were studied are listed in Table 2. The real-time PCR as well as the calculation of the relative expression level were done according to Betekhtin et al. [47].

## 5. Conclusions

In this work, we describe the early events of cell cycle activation during the germination and early stages of seedling development in the monocotyledonous model plant Brachypodium, which has also been proposed as a model to study grain dormancy in grasses.

1. Brachypodium embryos exhibit polysomaty, and nuclei with 2C, 4C, 8C, and 16C have been detected among embryo tissues. Nuclei with higher than 4C DNA content were found in the scutellum, coleorhiza, and coleoptile cells.
2. The cell cycle was initiated before radicle protrusion through coleorhiza and radicle elongation. Brachypodium embryo cells initiated DNA replication after only a few hours of imbibition and the first EdU-labelled nuclei were visible after 11 h of imbibition in the radicle tissues.

The results presented here can form the basis of future research not only on germination but also on the role of phytohormones and other germination stimuli such as ROS and NO in regulating seed dormancy in grasses as well as their role in cell cycle activation.

## Figures and Tables

**Table 1:** Percentage of nuclei in G1, S, and G2 phases in the cells of Brachypodium embryos during imbibition.

Imbibition Time (hours)	Nuclei (%)			4C/2C
	G1 (2C)	S	G2 (4C)	
4	57.1	16.7	26.11	0.46
6	52.57	20.76	26.67	0.51
8	53.9	16.8	26.29	0.49
10	56.46	21.51	31.03	0.55
11	50.92	21.01	28.07	0.55
12	51.48	23.18	25.34	0.49
13	52.72	15.54	31.74	0.60
14	46.77	24.24	28.98	0.62
15	47.74	18.39	33.87	0.71
16	45.91	23.42	30.67	0.67
17	n/a	n/a	n/a	n/a
18	38.72	24.77	36.51	0.94
19	38.92	22.72	38.36	0.99
20	38.57	20.4	41.03	1.06
21	41.64	17.99	40.37	0.97
22	40.85	20.73	38.42	0.94
23	42.66	19.6	37.74	0.88
24	41.28	21.73	36.99	0.90

**Table 2:** The oligonucleotide primers that were used for the RT-PCR reaction with the relevant description of the genes.

Gene Name	Gene Description	Primer Sequence (5′-3′)
AK437296	ubiquitin	pF-TCAAAATGCAAGAACGCAAA
		pR-TCCACACTCCACTTGGTGCT
Bradi1g54570.1	protein kinase binding	pF-TTGTGAAGAGGTTCGCGGATGC
		pR-CCTTCAAGCTCCTTCAGATCC
Bradi3g02270.1	cyclin-dependent kinase (CDK), subfamily CDKA	pF-CGAGAAGGTGGAGAAGATCG
		pR-CGATGGTCTCGTTGGTGTAG
Bradi4g25980.1	cyclin-dependent kinase (CDK), subfamily CDKB1	pF-AAGTGTACAAGGCGCAGGAC
		pR-ATCCCTTCGTCGTCCATCTC
Bradi3g40200.1	cyclin-dependent kinase (CDK), subfamily CDKB2	pF-AGGGCCAGACCATCCTCTAC
		pR-GGATCTTCTCGTGGTTCTGG
Bradi2g26510.1	cyclin-dependent kinase (CDK), subfamily CDKD	pF-ACAATGGCCAGACATGGTTT
		pR-CCATTGGAAACAATGAACGA
Bradi1g14820.1	CYCLIN, subfamily CYCA3	pF-ATCCTTGTTGACTGGCTCGT
		pR-CGGTCGATGTAGGAGATGGT
Bradi2g52760.1	CYCLIN, subfamily CYCB1	pF-GTCCTGGGAAAGCAGAAGGT
		pR-GGACGTTGACGACGTTGC
Bradi3g58300.1	CYCLIN, subfamily CYCD3	pF-AGCTGTGACTGCTTGCTCAT
		pR-GATAAGGTCAGACGAGCGGG
Bradi4g32556.1	CYCLIN-D4-1-RELATED	pF-CTTGTCTGTAGCGGCCAAGA
		pR-CTGGATCGTCATGGCTTCGA
Bradi3g03112.3	wee1-like protein kinase (WEE1)	pF-AGGATTTCTTCTGCACCCCG
		pR-GGAGATTTGGGGCAAGGGAT
